# Risk of SARS-CoV-2 Infection Breakthrough among the Non-Vaccinated and Vaccinated Population in Italy: A Real-World Evidence Study Based on Big Data

**DOI:** 10.3390/healthcare10061085

**Published:** 2022-06-10

**Authors:** Alessandro Perrella, Massimo Bisogno, Angelo D’Argenzio, Ugo Trama, Enrico Coscioni, Valentina Orlando

**Affiliations:** 1 Regional Task Force COVID-19, Campania Region, 80143 Naples, Italy; angelo.dargenzio@regione.campania.it (A.D.); ugo.trama@regione.campania.it (U.T.); coscionienrico@gmail.com (E.C.); 2 UOC Emerging Infectious Disease with High Contagiousness AORN Ospedali dei Colli P.O.C. Cotugno, 80131 Naples, Italy; 3 Sinfonia Regional Health Information System of Campania Region, 80143 Naples, Italy; massimo.bisogno@regione.campania.it; 4 Directorate-General for Health Protection, Campania Region, 80143 Naples, Italy; 5 Division of Cardiac Surgery, AOU San Giovanni di Dio e Ruggi d’Aragona, 84131 Salerno, Italy; 6 CIRFF, Center of Drug Utilization and Pharmacoeconomics, Department of Pharmacy, University of Naples Federico II, 80131 Naples, Italy

**Keywords:** SARS-CoV-2 infection, COVID-19, vaccine doses, COVID-19 symptoms

## Abstract

SARS-CoV-2 infection after vaccination can occur because COVID-19 vaccines do not offer 100% protection. The study aim was to assess duration of vaccination coverage, disease symptoms and type of hospitalization among non-vaccinated and vaccinated subjects to evaluate the vaccination trend over time. A retrospective cohort study was carried out among people testing COVID-19 positive in Campania Region using information from the Health Information System of Campania Region (Sinfonia). Vaccination status was assessed considering: no vaccination, partial vaccination and effective vaccination. Univariate and multivariate logistic regression models were constructed to evaluate the association between ICU admissions caused by COVID-19 and gender, age groups and vaccine type. Vaccine coverage duration trends were investigated using segmented linear regression and breakpoint estimations. Vaccination coverage was assessed by analyzing COVID-19 positive subjects in the 9 months after an effective dose vaccination. A significant risk of hospitalization in the ICU was caused by vaccination status: subjects non-vaccinated (OR: 7.14) and partially vaccinated (OR: 3.68) were 3 and 7 times more at risk of hospitalization, respectively, than subjects effectively vaccinated. Regarding subjects with an effective vaccination, the vaccine’s ability to protect against infection in the months following vaccination decreased. The risk of contracting COVID-19 after vaccination was higher 5 months (β = 1441, *p* < 0.001) and 7 months (β = 3110, *p* < 0.001) after administration of an effective dose. COVID-19 vaccines were demonstrated to protect from symptomatic infection by significantly reducing hospitalization risk, and their full protection against SARS-CoV-2 was demonstrated to decrease after 5 months regardless of age, gender or vaccine type.

## 1. Introduction

Since WHO declared the emergence of coronavirus disease 2019 (COVID-19) pandemic on 11 March 2020, over 5 million people have died worldwide, including over 130,000 people in Italy [1].

Due to the impact of severe acute respiratory syndrome coronavirus-2 (SARS-CoV-2) infection on the health systems of all countries, several countries and pharmaceutical companies have promoted research protocols to find a cure or develop a vaccine against SARS-CoV-2 [2,3,4].

To date, several vaccines based on different technologies have been produced and authorized. In Italy, at the end of 2020 and throughout 2021, the European and Italian authorities European Medical Agency (EMA) and Italian Medicines and Healthcare Products Regulatory Agency (AIFA) authorized the BNT162b2 mRNA (Pfizer–BioNTech) and ChAdOx1 nCoV-19 adenoviral (Oxford–AstraZeneca), CX-024414 mRNA (Moderna) and Ad26.COV2-S adenoviral (J&J) vaccines. Since the authorization of these vaccines, thanks to the extraordinary effort of all Italian healthcare workers as well as the civic responsibility of the Italian population, a high percentage of people received the COVID-19 vaccination. This vaccination campaign enabled a gradual decrease in infection across the country during the first 8 months of 2021 [5].

Since the marketing of new vaccines, the phenomenon known as “vaccine hesitancy” (VH) has spread, consisting of the delay in the acceptance or refusal of vaccines, despite the availability of services [6]. VH has recently been shown to be present in at least 15% of the world’s population, and even healthcare workers sometimes have doubts about vaccination [6]. A recent study evaluating VH in Italy investigated levels of COVID-19 vaccine risk perception and found that it was associated with a higher likelihood of being uncertain about attitudes towards the vaccination rather than rejection of the vaccine [7]. In Southern Italy, subjects showed a good level of knowledge of COVID-19 and its prevention and a fairly poor VH [8]. This attitude helped in the vaccination campaign in Italy, but while the inhabitants had confidence in the health system and underwent vaccination, the effectiveness and duration of vaccination coverage still seem to remain an uncertain issue and are not based on real big data. Despite more recent studies on the topic [9,10,11], there is still a lack of studies on vaccine efficacy based on real-world data. In fact, the risk of contracting COVID-19 despite having been vaccinated is not negligible, and there is a lack of real-world studies assessing vaccination coverage in the vaccinated and unvaccinated.

This study therefore aimed to assess the duration of vaccination coverage, disease symptoms and type of hospitalization among non-vaccinated and vaccinated subjects to evaluate the vaccination trend over time.

## 2. Materials and Methods

### 2.1. Data Sources: Sinfonia

The Regional Health Information System of Campania Region (Sinfonia) includes information on patient demographics for ~6 million residents, comprising a well-defined population in Italy (~10% of the population of Italy).

This data source was complete as it also included a data management system already validated in previous studies [12,13,14,15].

Sinfonia collects information, encrypted and anonymized, whose legal owner of the original data, in accordance with the privacy laws, is the local health unit (LHU). Basically, Sinfonia automatically compiles its big data from the electronic health records of local health units. Each citizen is recorded in a regional anagraphic database, and once the citizen has access to a laboratory test, the LHU system links the laboratory assay to the individual. After the results have been generated, they are stored in the LHU database. Therefore, all analyses of the data were carried out on encrypted and anonymized data using transparent data encryption protocols.

The aims of the Sinfonia tool [16] were as follows:

(1) Applying data science methods to big data in order to assess pandemic trends;
(2) Creation of predictive algorithms through AI methods;
(3) ML analysis, performed according to a Python scripting model (Spyder IDE 64bit ver), to perform predictive analysis on contagiousness.

In addition, during the pandemic emergency, Sinfonia was supplemented with all COVID-19-related data in order to create a tool to support health governance in managing the pandemic emergency. The characteristics of Sinfonia are described in Appendix A.

### 2.2. Study Design and Cohort Selection

A retrospective cohort study was carried out among people who tested positive for COVID-19 in Campania Region from 8 March 2021 to 31 October 2021. In total, 5,889,567 inhabitants of Campania Region in the study period were eligible for vaccination. All nasopharyngeal swabs tests performed among this reference population were collected by trained personnel of the regional healthcare system and/or authorized and trained territorial laboratory staff. The reverse transcription polymerase chain reaction (RT-PCR) test was performed with the use of a standardized RT-PCR machine from Coronavirus Network Laboratory (CoroNetLab); four genes were analyzed, namely the RdRP, S and N genes specific to SARS-CoV-2 and the E gene, and results were expressed as the cycle threshold (Ct). A Ct value of less than 30, which indicated an increased viral load, was used to determine infectivity [17,18].

Test results were considered fully positive when all 4 genes were amplified by Rt-PCR, while in all other cases the results were considered doubtful and repeated [17,18]. Participant consent was mandatory and was requested from all participants in order to receive SARS-CoV-2 PCR test results before or after vaccination. All participants who tested positive were monitored until the first negative PCR test. Any previous SARS-CoV-2 infection was evaluated according to serology or a previous nasal swab testing positive already recorded in Sinfonia. Patients who had already recovered from a previous SARS-CoV-2 infection were excluded from the analysis.

Data on clinical symptoms were collected for all individuals who tested positive for the nasal swab, according to the Italian National Institute of Health. Typical COVID-19 symptoms were fever, cough, or change or loss of taste or smell. Participants were recorded as suffering from other symptoms if reporting one of the following: shortness of breath, sore throat, runny nose, headache, muscle aches, extreme fatigue, diarrhea, nausea or vomiting, or small itchy red patches on fingers or toes. All these symptoms had an onset date within 14 days before or after the PCR positive sample date.

Data extraction from Sinfonia was carried out every month in order to obtain regular reports of vaccine/positive trends until the final analysis on 31 October 2021. All collected data, after using the ML algorithm, were anonymized and encrypted according to transparent data encryption.

**Figure 1:**
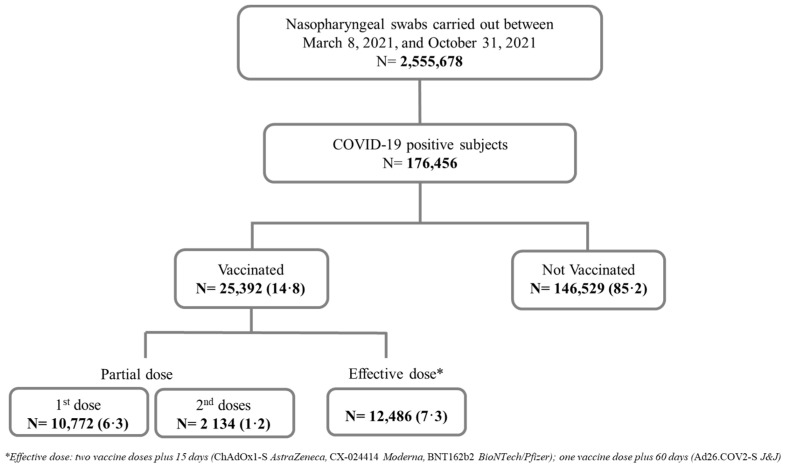
Flow chart: cohort selection.

Briefly, an ML-based algorithm was used in data mining on Sinfonia to match records from the SARS-CoV-2 RT-PCR nasal swab test and the status of vaccination daily according to the following timetable:

○ Positive subjects without vaccination were named “non-vaccinated”;
○ Positive subjects who received the 1st vaccine dose (<15 or >15 days) or two vaccine doses plus <15 days were named “partially vaccinated”;
○ Positive subjects who received two vaccine doses plus >15 days since the second dose of BNT162b2 mRNA (Pfizer–BioNTech), ChAdOx1 nCoV-19 adenoviral (AstraZeneca) and CX-024414 mRNA (Moderna) or 60 days after one shot in the case of Ad26.COV2-S adenoviral (J&J) were defined as “effectively vaccinated”. Figure 1 shows the flow chart of cohort selection.

All subjects provided written informed consent to vaccination and data storage on a big data system collecting all COVID-19 patients’ data and related clinical history (symptoms, hospital admission and related follow-up, previous clinical status) according to European Privacy Policy to manage the pandemic.

Time elapsed from the second dose and onset of COVID-19 was calculated for all individuals to evaluate the risk of infection in a time-dependent way. In addition, once a positive subject was recognized among those vaccinated, it was assessed according to the number of days since the vaccine.

### 2.3. Outcomes

The primary outcome of the present study was to assess the effective COVID-19 vaccine coverage by measuring differences in the risk of contracting severe COVID-19 with hospitalization in the intensive care unit (ICU) among subjects effectively vaccinated or non-vaccinated. The secondary outcome was to evaluate the duration of vaccination coverage over time, stratifying the information by age group and vaccine type.

### 2.4. Statistical Analyses

The study population baseline characteristics were analyzed using descriptive statistics. Quantitative variables were described as counts and percentages. The chi-square test and *t*-test were performed to determine the difference between non-vaccinated and vaccinated subjects who tested positive for COVID-19. In particular, vaccinated subjects were categorized into two groups: those partially vaccinated who received one vaccine dose or two vaccine doses, and those effective vaccinated who received two vaccine doses plus 15 days: BNT162b2 mRNA (Pfizer–BioNTech), ChAdOx1 nCoV-19 adenoviral (AstraZeneca) and CX-024414 mRNA (Moderna), or one vaccine dose plus 60 days in the case of Ad26.COV2-S adenoviral (J&J) administration.

Univariate and multivariate logistic regression models were constructed to evaluate the association between intensive care unit (ICU) admissions, gender, age groups (i.e., 40–59 years, 60–79 years and ≥80 years vs. 0–39 years) and vaccine type (no vaccination and partial vaccination vs. effective vaccination).

To determine the vaccination coverage among individuals who received effective vaccination, the trend over time was explored using segmented linear regression models and breakpoint estimates. Breakpoints were identified by testing for differences in the slope and intercepts of the trend and then several linear models were implemented. Changes in the slope segment indicated an impact of vaccination coverage on protection against COVID-19 infection. Every linear model was expressed as follows: yt = a + b ∗ t + et, where a is the intercept, b is the slope and et is the error term. Coefficients (β) were considered statistically significant with a *p* value < 0.05. The 95% confidence intervals (CIs) for each breakpoint were also obtained.

In addition, vaccination coverage was assessed by analyzing the trend in the percentage of COVID-19 positive subjects in the 9 months after an effective vaccination stratified by age group and vaccine type. Statistical analyses were performed using the R platform (version 3.6, The R Formulation for Statistical Computing, Vienna, Austria).

## 3. Results

During the study period, 8 March 2021 to 31 October 2021, in Campania Region, 2,555,678 nasal swabs were performed among subjects aged 18–98 years on a total of 5,889,567 inhabitants. Overall, among the COVID-19 positive subject cohort, 85.2% were non-vaccinated (N = 146,529) and 14.8% (N = 25,392) were vaccinated (Figure 1). Of 25,392 subjects who received at least one vaccine dose, 7.5% (N = 12,906) received a partial vaccination and 7.3% (N = 12,486) received an effective vaccination.

Among the 171,921 subjects who tested COVID-19 positive, 51.2% were females.

The analysis stratified by age group showed that 50.9% were aged 0–39 years, 29.4% 40–60 years, 16.3% 60–79 years and 3.4% were aged more than 80 years.

Disease symptom-related data were not available for 34,119 subjects (19.8%) included in the analysis.

Particularly, the percentage of subjects with severe or critical (deceased) symptoms decreased in the cohort of vaccinated subjects compared to non-vaccinated subjects. Among a total of 482 subjects with severe symptoms, 89.4% of the subjects were non-vaccinated, 7.5% of the subjects were vaccinated with a partial dose and 3.1% of the subjects were effectively vaccinated.

Similarly, out of 57 subjects with critical symptoms (deceased), 82.5% of the subjects were non-vaccinated, 10.5% were vaccinated with a partial dose and 7.0% were vaccinated with an effective dose.

Moreover, 2.7% of COVID-19 positive subjects were hospitalized and 0.1% were admitted to an intensive care unit.

Among hospitalized subjects, the majority (83.7%) were non-vaccinated, 10.3% received a partial dose and 6.0% received an effective dose.

Among subjects hospitalized in intensive care, the majority (90.5%) were non-vaccinated, 7.6% received a partial dose and 1.9% received an effective dose (Table 1).

Table 2 shows the results of univariate and multivariate logistic regression analyses, which revealed that three independent variables contributed statistically significantly to the model: the subjects’ gender, age and vaccination status. These variables were found to be the main determinants of the risk of admission to the intensive care unit caused by COVID-19.

A significant association was found between the risk of ICU admission and gender. Males (adjusted odds ratio (OR): 1.70; 95% CI: 1.29–2.24, *p* value < 0.001) had twice the risk of admission to intensive care compared to females. A similar trend was found in the case of the age variable. Subjects aged 60–79 years (adjusted odds ratio (OR): 33.53; 95% CI: 19.31–58.23, *p* value < 0.001) and those aged more than 80 years (adjusted odds ratio (OR): 29.04; 95% CI: 14.48–58.27, *p* value < 0.001) were more likely be hospitalized compared to those aged 0–39 years. Regarding the vaccination status, subjects with partial vaccination (adjusted OR: 3.68; 95% CI: 1.23–11.02, *p* value < 0.001) and non-vaccinated subjects (adjusted OR: 7.14; 95% CI: 2.64–19.27, *p* value < 0.001) were more at risk of ICU admission than those who received an effective vaccination.

In Campania Region, from 8 March 2021 to 31 October 2021, 3,699,683 subjects received a complete vaccine schedule, with a vaccine acceptance rate of 63%. Among the population vaccinated with an effective dose, 0.33% (N = 12,486) contracted COVID-19.

**Figure 2:**
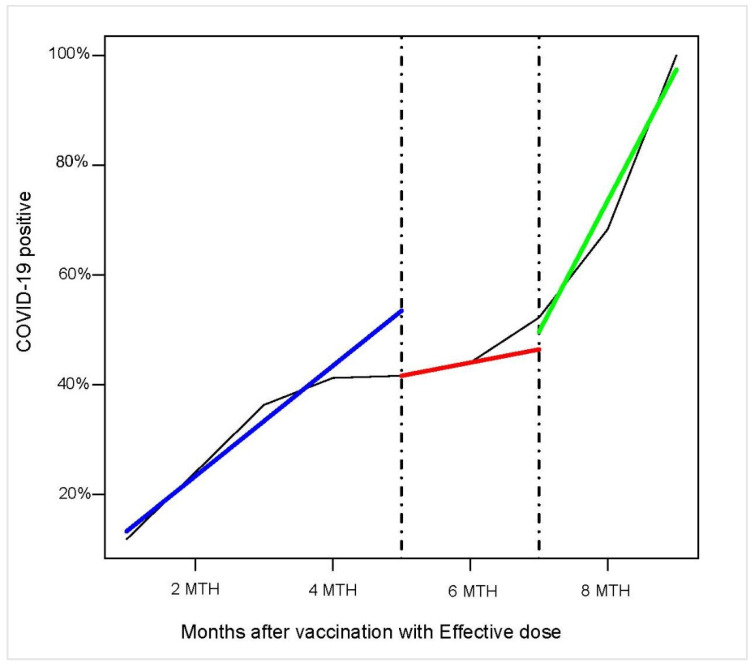
Segmented linear regression models. Notes: Segmented linear regression model and breakpoint estimates determined vaccination coverage in subjects who received effective vaccination and changes in trend over time. Changes in the slope segment indicated an impact of vaccination coverage on protection against COVID-19 infection.

The vaccine coverage duration in the months following the administration of an effective dose was investigated through the estimation of breakpoints, i.e., points in which data show deviations from stability in the background trend. Figure 2 shows the trend in the percentage of COVID-19 positive subjects in the 9 months following effective vaccination. Two breakpoints were identified from the analysis: the first point was five months after vaccination (β = 1441, *p* < 0.001); the second point was seven months after vaccination (β = 3110, *p* < 0.001).

**Figure 3:**
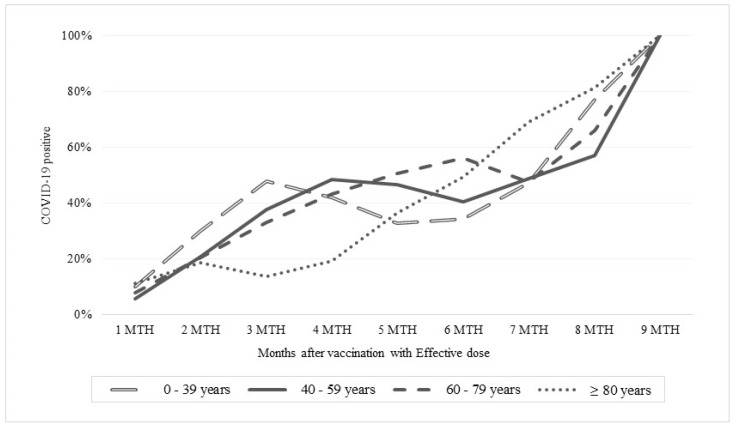
Percentage of COVID-19 positive patients stratified by number of months after an effective vaccination and stratified by age groups.

The analysis stratified by age group showed a similar trend in subjects aged ≤79 years in terms of increased number of COVID-19 positive patients (about 50%) up to the sixth month after vaccination. On the contrary, among subjects aged over 80 years, the trend up to the sixth month after vaccination was different: the percentage of positive subjects did not exceed 40%. On the other hand, six months after vaccination, the trend was similar in all age groups (Figure 3).

**Figure 4:**
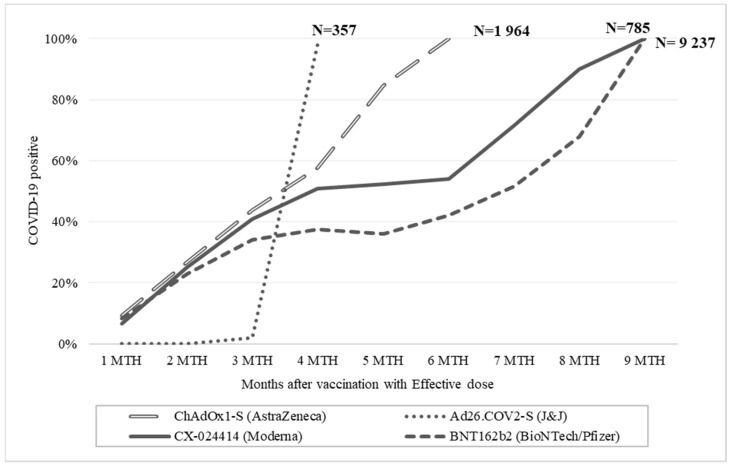
Percentage of COVID-19 positive patients stratified by number of months after an effective vaccination and stratified by age groups.

Moreover, the analysis stratified by vaccine type revealed that all subjects vaccinated with Ad26.COV2-S adenoviral (J&J) (n = 357) tested positive for COVID-19 within one month after the effective vaccination (third month). On the other hand, 50% of the subjects vaccinated with ChAdOx1 nCoV-19 adenoviral (AstraZeneca) (n = 1964) tested positive from the fourth month after vaccination until they were all positive by the sixth month after vaccination. Around half of the subjects vaccinated with CX-024414 mRNA (Moderna) (n = 785) tested positive in the fourth month after vaccination, while the trend remained constant, between the fourth and sixth months and then increased to 90% in the eighth month after vaccination. Finally, 37% of the subjects vaccinated with BNT162b2 mRNA vaccine (Pfizer–BioNTech) (n = 9237) tested positive from the fourth month after vaccination; the trend remained constant between the fourth and fifth month and then increased to 67% in the eighth month after vaccination (Figure 4).

## 4. Discussion

Vaccines have been a very successful technology for controlling infectious diseases in the past. COVID-19 has represented a never-before-experienced global emergency with worldwide spread and high mortality rate, and therefore vaccines have represented a possible solution to stop SARS-CoV-2 from spreading. Indeed, since the early phase of the vaccine campaign, COVID-19 contagiousness has registered a decrease [5]; however, few real-world studies are available on prolonged follow-up and on larger cohort populations [19]. As a first consideration, according to our primary outcome, the results of this large community study based on the Campania Region population (ISTAT census citizens in December 2020: 5,889,567) showed that vaccination with two doses of BNT162b2 or ChAdOx1 still significantly reduces the risk of new PCR-positive SARS-CoV-2 infection.

In addition, it is worth noting that vaccination demonstrated a highly significant reduction in ICU admission (Table 2). Despite this, results revealed an infection rate of 7.3% among the population that received an effective vaccination, compared to 0.3% of the total vaccinated population in the Campania Region. The risk of contracting COVID-19 after vaccination was high 2 months after the administration of a partial dose and 5 months after the administration of an effective dose. The highest proportion of infected positives was asymptomatic while the dynamics of protection varied by vaccine type, with initially similar efficacy of both mRNA and ChAdOx1 vaccines becoming less effective after 4 months with a more rapid decline in coverage for the vector-based adenoviral vaccine. The Ad26.COV2-S vaccine had a shorter duration of vaccination coverage than the others (one month after completion of the vaccination program). Those findings, however, demonstrated that vaccines are able to decrease the severity of SARS-CoV-2 disease once infected. Nonetheless, it seems also clear that vaccines, even if effective for decreasing the severity of disease and risk of severe hospitalization, showed a decrease in their efficacy in protecting against SARS-CoV-2 contagiousness throughout the time in a significant percentage but not the majority of vaccinated people. This consideration would suggest that, at least in some cases, the vaccine protected against symptomatic disease but not against infection. Some explanations could be mainly viral and immune-related. Indeed, according to the available data of the Italian National Institute of Health, in Italy, and therefore in the Campania Region, there was an increase in the frequency of the Delta variant in Italy, which by the end of July had reached almost 95% of all viruses isolated [20]. Therefore, the dominance of the Delta variant concomitant with the progress of the vaccination campaign in Italy may have played a crucial role in this trend reversal. Another explanation could be in different effects of vaccination on immunity, cellular or humoral, possibly determining different infective states [21] mainly characterizing asymptomatic subjects. These considerations also underline the need to better understand what kind of impact asymptomatic vaccinees may have on the spread of SARS-CoV-2. Indeed, even if the current findings of vaccine effectiveness to protect against severe outcomes would seem to suggest that virus transmission and nasopharyngeal viral presence may have limited consequences, we could have some important consequences over time. In fact, in absence of universal vaccination, possible environments where the SARS-CoV-2 may develop elusive strategies by increasing its mutational rate or fitness could compromise vaccine efficacy. The latter event could make herd immunity less likely with possible severe evolution in particular settings of patients. Although this could be a possible future scenario, our findings may represent useful evidence for preventing this phenomenon. Particularly, the present study has two main strengths. First, it provides extensive documentation on a large cohort of breakthrough infections, based on regional big data where all COVID-19 positive data are automatically evaluated, matched and analyzed for their vaccine status by a real-time ML algorithm, minimizing error in records and giving a real-time scenario and trend. Second, the studied cohort is one of the largest presented in literature and represents all ages that underwent vaccination with a very good representation of all currently approved vaccines. Moreover, the study found that although the current approved COVID-19 vaccines are extremely effective in reducing hospitalization and particularly ICU admissions, breakthrough infections occur with a breakpoint between the 5th and 7th months after vaccination, and they may carry a potential infectiveness. This event could represent a challenge since such infections are often asymptomatic and may pose a risk to vulnerable populations. Consequently, a boost dose could be a possible strategy while awaiting the antiviral [22,23] that could give us a final weapon against SARS-CoV-2. However, considering the highest percentage of asymptomatic patients and the limited data about their capacity to transmit SARS-CoV-2, further screening, quarantine procedures and other prevention strategies should be guaranteed in all vaccinated subjects. The main strength of this paper is the high number of evaluated subjects. This was possible thanks to big data mining. However, this would also be a possible limitation since the study covers several months; therefore during the retrospective evaluation period, not all of the Campania Region was assessed, for previous infection with serology or nasal swab RT-PCR. Further, possible interference could be related to the time elapsed in the heterologous vaccination, particularly the time elapsed between the two vaccine inoculations, which was not the same for all individuals. Another limitation of the study is related to the characteristics of the sample, which, although very large, at the time of data mining through our ML algorithm, does not take into account whether severe infections or deceased may be due to co-existing diseases, as we do not have data on clinical conditions and comorbidities. Finally, it is possible that asymptomatic cases may have been missed, i.e., those who could have had an asymptomatic infection between the testing period and therefore tested negative once screened. This factor may have led to an underestimation of the difference in vaccinated individuals who contracted COVID-19.

## 5. Conclusions

This study confirmed the evidence to date by demonstrating in a real-world setting that COVID-19 vaccines protect from symptomatic infection by significantly reducing hospitalization risk. However, findings showed that the full protection against SARS-CoV-2 granted by COVID-19 vaccines tends to decrease after five months regardless of age, gender or type of vaccine. This is the first study to examine an entire real population such as the Campania Region, which represents 10% of the Italian population, relating the duration and vaccination coverage in the risk of contracting COVID-19 to the number of vaccine doses received. Real-world-based results obtained by testing the hypotheses on an entire population suggest an incentive, on the citizen’s side, to trust in vaccination campaigns as well as to consider that vaccination coverage decreases in the following months. On the healthcare system side, the results of this study are intended to provide real-world data to aid future healthcare decisions regarding the COVID-19 pandemic. Indeed, in a future perspective, an approach based on big data to investigate real-world evidence related to vaccine efficacy and safety could also help to integrate information on populations normally excluded from clinical trials, particularly those subjects on whom there are no robust data on the efficacy of medicines until after they have been placed on the market. The impact of real-world evidence, deriving from a planned and rigorous observation of big data, is crucial in healthcare planning. A surveillance approach based on the use of an integrated big data system to match all clinical conditions offers precise and real analysis with a low incidence of errors in the categorization of subjects. Therefore, although it is a retrospective analysis, it strongly suggests that an approach based on ML and AI should be considered in a pandemic situation. Further, this paper also shows that a big-data-based strategy with a centralized data harvest may be useful in healthcare management not only to understand the efficacy and safety of a treatment schedule but also as a possible tool to improve a preventive medical approach and optimize pharmaceutical expenditure through an innovative healthcare governance tool.

## Figures and Tables

**Table 1:** General characteristics of COVID-19 positive patients.

	Total N (%)	No Vaccination	Partial Vaccination N (%)		Effective Vaccination *
			1st Dose	2nd Doses	
	171,921 (100)	146,529 (85.2)	10,772 (6.3)	2134 (1.2)	12,486 (7.3)
**Gender**					
Male	83,924 (48.8)	71,693 (85.4)	5249 (6.3)	1168 (1.4)	5814 (6.9)
Female	87,997 (51.2)	74,836 (85.0)	5523 (6.3)	966 (1.1)	6672 (7.6)
**Age Groups**					
0–39 years	87,464 (50.9)	79,686 (91.1)	3101 (3.5)	1099 (1.3)	3578 (4.1)
40–59 years	50,539 (29.4)	42,042 (83.2)	3419 (6.8)	462 (0.9)	4616 (9.1)
60–79 years	28,079 (16.3)	21,331 (76.0)	3371 (12.0)	372 (1.3)	3005 (10.7)
≥80 years	5839 (3.4)	3470 (59.4)	881 (15.1)	201 (3.4)	1287 (22.0)
**Vaccine type ^#^**					
ChAdOx1-S (AstraZeneca)	6067 (3.5)	-	3913 (64.5)	190 (3.1)	1964 (32.4)
Ad26.COV2-S (J&J)	1181 (0.7)	-	824 (69.8)	-	357 (30.2)
CX-024414 (Moderna)	1774 (1.0)	-	898 (50.6)	91 (5.1)	785 (44.3)
BNT162b2 (Pfizer–BioNTech)	15,657 (9.1)	-	5422 (34.6)	998 (6.4)	9237 (59.0)
**Disease Symptoms**					
Asymptomatic	112,251 (65.3)	95,482 (85.1)	7343 (6.5)	1526 (1.4)	7900 (7.0)
Subclinical	14,339 (8.3)	12,542 (87.5)	865 (6.0)	134 (0.9)	798 (5.6)
Mild	10,673 (6.2)	9368 (87.8)	599 (5.6)	60 (0.6)	646 (6.1)
Severe	482 (0.3)	431 (89.4)	35 (7.3)	1 (0.2)	15 (3.1)
Deceased	57 (0.03)	47 (82.5)	6 (10.5)	-	4 (7.0)
Not available	34,119 (19.8)	28,659 (84.0)	1924 (5.6)	413 (1.2)	3123 (9.2)
**Hospitalization**					
Yes	4705 (2.7)	3939 (83.7)	431 (9.2)	52 (1.1)	283 (6.0)
No	167,216 (97.3)	142,590 (85.3)	10,341 (6.2)	2082 (1.2)	12,203 (7.3)
**Intensive Care Unit (ICU)**					
Yes	211 (0.1)	191 (90.5)	16 (7.6)	-	4 (1.9)
No	171,710 (99.9)	146,338 (85.2)	10,756 (6.3)	2134 (1.2)	12,482 (7.3)

* Effective vaccination: two vaccine doses plus 15 days (ChAdOx1-S, CX-024414, BNT162b2); one vaccine dose plus 60 days (Ad26.COV2-S). ^#^ Vaccine type: only for subjects who had received at least one dose of vaccine.

**Table 2:** Univariate and multivariate logistic regression of the risk of hospitalization in intensive care unit (ICU) caused by COVID-19.

	Unadjusted OR (95% CI)	*p*-Value	Adjusted OR (95% CI)	*p*-Value
**Gender**				
Male (vs. Female)	1.59 (1.20–2.09)	0.001 *	1.70 (1.29–2.24)	<0.001 *
**Age groups**				
40–59 years (vs. 0–39 years)	5.57 (3.05–10.14)	<0.001 *	5.99 (3.28–10.90)	<0.001 *
60–79 years (vs. 0–39 years)	29.73 (17.13–51.57)	<0.001 *	33.53 (19.31–58.23)	<0.001 *
≥80 years (vs. 0–39 years)	20.39 (10.21–40.69)	<0.001 *	29.04 (14.48–58.27)	<0.001 *
**Vaccination**				
Partial Vaccination (vs. Effective Vaccination)	3.94 (1.31–11.79)	0.014 *	3.68 (1.23–11.02	0.020 *
No Vaccination (vs. Effective Vaccination)	4.11 (1.52–11.06)	0.005 *	7.14 (2.64–19.27)	<0.001 *

Abbreviations: CI, confidence interval; OR, odds ratio. * *p*-value < 0.05 was considered to be statistically significant.

**Figure A1:**
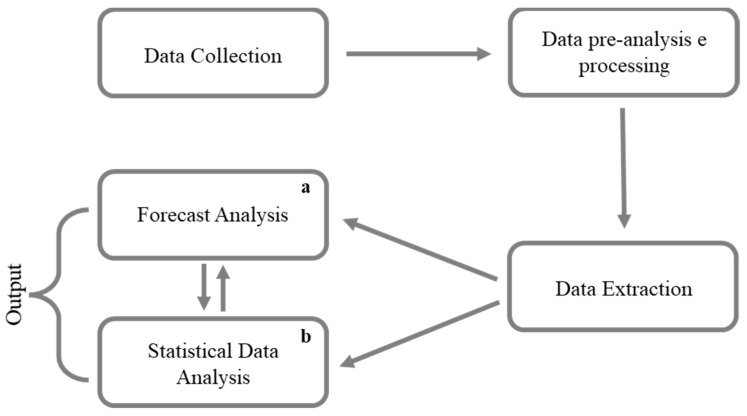
Block diagram of the machine-learning-based algorithm extraction for data mining for time series forecasting and statistical analysis. The current paper shows findings related to output box b.

## Data Availability

Permission to use anonymized data for this study was granted to the researchers of Regional Direction for Health Management, Pharmaceutical Unit. Requests for information on data access can be directed to the corresponding author.

## Appendix A. Description of Machine Learning Process

ML analysis was performed according to a Python scripting model (Spyder IDE 64bit ver) to select, extract and match individuals vaccinated/negative or vaccinated/positive and to perform forecasting analysis on contagiousness and trend in coverage. Obtained data were further organized on Tableau Professional.

Briefly, machine learning algorithms take the information that represents the relationship between items in data sets and export them on a second software for statistical analysis or graphical representation. Further, they create models to forecast future trends and results. These models are nothing more than actions that will be taken by the machine to achieve a result. This approach considers three main elements: Event I (positive or negative Test), CategoI(c) (Type of Time of subject, vaccinated or unvaccinated and Time (t) according to the following formula for a trend evaluation: Time series (Ts) = (Ep/En + Cv/Cu). Therefore, data mining was automated according to a machine learning algorithm as shown in Figure A1.
